# Risk of mortality among inpatients with COVID‐19 and type 2 diabetes: National data from Kuwait

**DOI:** 10.1002/edm2.287

**Published:** 2021-07-10

**Authors:** Ebaa Al‐Ozairi, Rosemary Brown, Yasmine Hamdan, Lulwa Alabdullah, Nia Voase, Jumana Al Kandari, Dalal Alsaeed, Abdulla Al Ozairi, Amal Hasan, Fahd Al‐Mulla, Srinivasa Vittal Katikireddi, Stuart R. Gray, Jason M. R. Gill, Carlos A. Celis‐Morales, Naveed Sattar, Paul Welsh

**Affiliations:** ^1^ Clinical Research Unit Dasman Diabetes Institute Dasman Kuwait; ^2^ Department of Medicine Faculty of Medicine Kuwait University Kuwait City Kuwait; ^3^ Institute of Cardiovascular and Medical Sciences University of Glasgow Glasgow UK; ^4^ Ministry of Health Kuwait City Kuwait; ^5^ Department of Psychiatry Faculty of Medicine Kuwait University Kuwait City Kuwait; ^6^ Department of Genetics and Bioinformatics Dasman Diabetes Institute Dasman Kuwait; ^7^ Public Health Institute of Health and Wellbeing University of Glasgow Glasgow UK

**Keywords:** COVID‐19, death, diabetes

## Abstract

**Introduction:**

To investigate type 2 diabetes as a risk factor for COVID‐19 death following hospital admission in Kuwait.

**Methods:**

A retrospective cohort study using data from a central hospital that cared for all hospitalized COVID‐19 patients in Kuwait. We investigated the association between type 2 diabetes, with COVID‐19 mortality using multiply imputed logistic regression and calculated the population attributable fraction.

**Results:**

A total of 5333 patients were admitted with COVID‐19, of whom 244 died (4.6%). Diabetes prevalence was 24.8%, but 53.7% of those who died had diabetes. After adjusting for age, sex, ethnicity and other comorbidities, diabetes was associated with death (OR 1.70 [95% CI 1.23, 2.34]) and admission to the intensive care unit more than 3 days after initial admission (OR 1.78 [95% CI 1.17, 2.70]). Assuming causality, the population attributable fraction for type 2 diabetes in COVID‐19 death was 19.6% (95% CI 10.8, 35.6).

**Conclusion:**

Type 2 diabetes is a strong risk factor for COVID‐19 death in the Middle East. Given the high prevalence of type 2 diabetes in the Middle East, as well as many Western countries, the public health implications are considerable.

## Graphical abstract

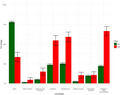

## 1 INTRODUCTION

The COVID‐19 pandemic^1^ has led to efforts to identify people at greatest risk including older age groups, those with comorbidities such as type 2 diabetes and cardiovascular disease (CVD), poor oxygen saturation and elevated markers of inflammation.^2^, ^3^, ^4^ The association of type 2 diabetes with poor outcomes is of particular interest,^5^ since it is a risk factor that is potentially amenable to public health interventions.

Kuwait faces specific challenges in terms of health care, due to the high burden of obesity, poor lifestyle and type 2 diabetes. Specifically, prevalence of type 2 diabetes in older adults is around 25% and obesity 40%–50%.^6^, ^7^ Previous studies and a systematic review have shown that the issue of incidence and clinical management of type 2 diabetes in Kuwait and the Middle East in general is complicated by several lifestyle and cultural circumstances including low levels of habitual physical activity, peer and family pressure to consume a poor diet and poor medication adherence.^8^ For instance, a hot climate, as well as lack of facilities and infrastructure to promote physical activity (such as pavements and walking routes), high traffic volume and cultural expectations surrounding PA behaviour among women are highly impactful.^9^ This raises the question of whether diabetes is a particular risk factor for poor COVID‐19 outcomes in the Middle East, since the severity and prevalence of type 2 diabetes is distinct in this area of the world. One recent study specifically in people with type 2 diabetes in Kuwait suggests that diabetes patients with COVID‐19 have poor clinical prognosis.^10^

We have previously reported demographic and clinical data on the first consecutive 1096 patient to be admitted to hospital with COVID‐19 in Kuwait,^11^ and here, we expand to make use of this resource. Specifically, our objective was to investigate the importance of type 2 diabetes as a risk factors for COVID‐19 death in Kuwait, compared with the general population and report the population attributable fraction (PAF) for type 2 diabetes.

## 2 METHODS

### 2.1 Study population

This retrospective cohort study included a single cohort of adult and paediatric inpatients admitted to Jaber Al Ahmad Al Sabah hospital, the largest tertiary hospital with 1240 bed capacity, and has been named the dedicated COVID‐19 hospital by Ministry of Health for Kuwaiti residents and visitors with diagnosis of COVID‐19 based on a positive PCR testing. Mandatory health insurance is required for all expatriates in Kuwait and is enforced through residency requirements. The present cohort included those who were admitted between 24/02/20 and 15/07/20. In line with strengthening the reporting of observational studies in epidemiology (STROBE) guidance, as data were obtained through routinely collected available data, no post hoc power calculation has been performed. All patients with a positive RT‐PCR test were admitted to Jaber Al Ahamd Al Sabah hospital. All patients in this study had a confirmed diagnosis of COVID‐19 based on a positive result of RT‐PCR assay of nasal and/or nasal and pharyngeal swabs, in accordance with WHO interim guidance. The data are derived from in‐hospital databases used during routine care as previously described.^11^ Ethical approval for use of the data was obtained by the standing committee for coordination of health and medical research in the ministry of health in Kuwait (IRB 2020/1400).

### 2.2 Outcome of interest

The primary outcome was death from COVID‐19, from the death certificate, which was matched to patients' hospital file numbers and study numbers using the Kuwaiti national Civil ID number, a unique number given to all residents. A secondary outcome was transfer to the intensive care unit (ICU) during COVID‐related hospitalization; this secondary outcome was only explored in patients who were not admitted to intensive care in the first 3 days following general admission.

### 2.3 Exposures of interest

Exposures of interest included age, sex, nationality and ethnicity, smoking status and clinical comorbidities, including type 2 diabetes. Vitals taken on admission included temperature, pulse, respiratory rate and oxygen saturation, systolic and diastolic blood pressure and body mass index. Laboratory biomarkers variables considered in the present study included routinely measured (as standard of care) platelet count, white blood cell count, estimated glomerular filtration rate (eGFR) and liver functions tests (gamma‐glutamyl transferase [GGT], alanine transaminase [ALT] and aspartate aminotransferase [AST]) as well as C‐reactive protein (CRP) and urea. Clinical vitals and biomarkers of interest were measured within seven days of admission.

### 2.4 Statistical analyses

Associations between exposures and outcomes of interest were first explored using a complete case analysis. All tests were performed in R using version 4.0.2. We then implemented and reported an analysis based on multiple imputations by chained equations (MICE) as part of the ‘mice’ R package.^12^ The imputation methods were predictive mean matching for continuous variables and proportional odds models for ordered factor variables. The number of iterations for each permutation was 10, with 10 permutations performed. Convergence was checked using plots. Pooled confidence intervals were derived using Wald method with pooled estimates and SEs. We reported two models, the first including age, sex, ethnicity and baseline comorbidities. The second model also included clinical vitals and admission and biomarkers of interest. Logistic regression using the multiple imputation data sets was implemented, using model development as detailed above. Additionally, for the first, simpler, model for the mortality outcome, analyses were stratified by median age (43 years) and sex. PAFs were derived for comorbidities using model 1, as above, using the ‘AF’ R package.^13^

## 3 RESULTS

**FIGURE 1:**
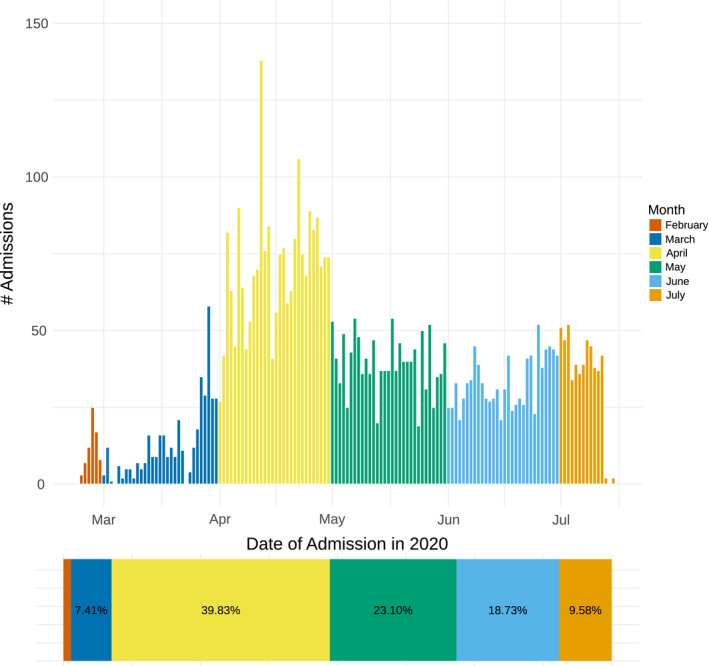
Temporal pattern of admission to Jaber Al Ahmad Al Sabah hospital for 5333 patients with PCR positive COVID‐19 between 24/02/20 and 15/07/20

**FIGURE 2:**
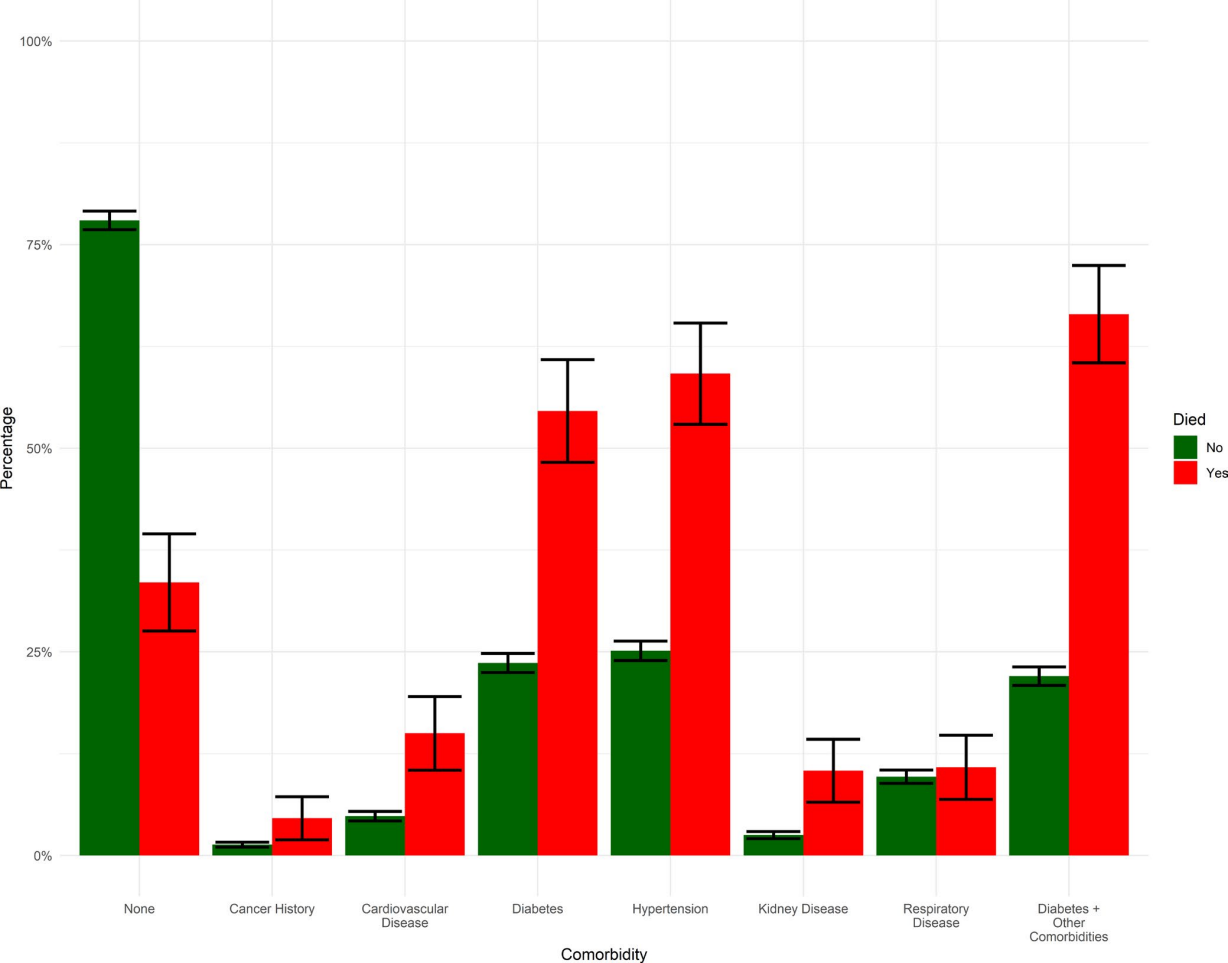
Proportion of patients with selected comorbidities, separately by COVID‐19 death status

Between 24/02/20 and 15/07/20, 5333 patients were admitted to hospital with confirmed COVID‐19. Nearly, two thirds of admission occurred between April and May 2020 (Figure 1). Patients admitted to hospital had mean age 44.2 (standard deviation [SD] 17.2) and were more often male sex (64.3%). Patient nationality was Kuwaiti in 55.8% of patients, and ethnicity was Arab in 67.1% and South Asian in 31.0%. Type 2 diabetes was diagnosed in 1317 patients (24.8%) and hypertension in 1417 (26.7%). Of the 5333 patients who were admitted, 244 died (4.6%). Type 2 diabetes prevalence in hospitalized patients was 24.8%, but 53.7% of those who died had type 2 diabetes (Figure 2). In addition, COVID‐19 death was more common in older patients (those who died were 15 years older than those who survived) in men and non‐Kuwaitis (Table 1).

**TABLE 1:** Demographics and comorbidities by COVID‐19 death status

Demographics	Alive 5089 (95.4%)	Died 244 (4.6%)
Male sex	3243 (63.8%)	187 (76.6%)
Age (years)	43.5 (17.1)	58.8 (13.4)
Nationality		
Kuwaiti	2851 (56.4%)	107 (44.0%)
Other (40 Nationalities + ‘Other’)	2205 (43.6%)	136 (56.0%)
Missing	33	1
Ethnicity		
African/Black	27 (0.5%)	1 (0.4%)
Arab	3410 (67.5%)	143 (58.6%)
Caucasian	60 (1.2%)	4 (1.6%)
East Asian	6 (0.1%)	1 (0.4%)
South Asian	1547 (30.6%)	95 (38.9%)
Missing	39	0
Type 2 diabetes	1186 (23.4%)	131 (54.6%)
Cardiovascular disease	245 (4.8%)	36 (15.0%)
Cancer history	68 (1.3%)	11 (4.6%)
Hypertension	1275 (25.1%)	142 (59.2%)
Kidney disease	128 (2.5%)	25 (10.4%)
Respiratory disease	491 (9.7%)	26 (10.8%)

Of the 4860 patients at risk (excluding 473 patients who were admitted to ICU within 3 days of general admission), 133 (2.7%) progressed to ICU admission. Of those admitted to ICU, 51.9% had type 2 diabetes.

In multivariable models including simple demographics and comorbidities, age was strongly independently associated with death, as was male sex and South Asian ethnicity (Table 2). Comorbidities associated with death were type 2 diabetes, cancer, cardiovascular disease and renal disease; the association for type 2 diabetes was strong (OR 1.70). There was no interaction between ethnicity, or other comorbidities, and type 2 diabetes in the association with death. Stratifying separately by age and sex found stronger associations between type 2 diabetes and mortality in those aged ≤43 years (OR 2.70 vs 1.59; *P*‐for interaction = .0178) and females (OR 3.57 vs 1.40; *P*‐for interaction = .16) (Table 3). In the second adjustment model, type 2 diabetes was not associated with death after adjusting for additional clinical and laboratory variables (low oxygen saturation, high urea, high CRP, high neutrophil count, liver function tests (AST, ALT, GGT), eGFR and low platelet count) (OR 1.08; 95% CI 0.71–1.66). Risk associations were generally weaker for ICU admission, but type 2 diabetes remained a significant risk factor (Table 2).

**TABLE 2:** Multivariable demographic and comorbid predictors of COVID‐19 death and ICU admission among COVID‐19 admissions in Kuwait

	Death from COVID‐19 *N* = 5333, *n* deaths =244	ICU admission (after 3 days) *N* = 4860, *n* admissions =133
	Odds ratio (95% CI)	Odds ratio (95% CI)
Age (years)	1.05 (1.04, 1.06)	1.05 (1.04, 1.06)
Sex		
Female (ref)	1.00	1.00
Male	2.04 (1.46, 2.84)	1.33 (0.91, 1.95)
Ethnicity		
Arab (ref)	1.00	1.00
South Asian	3.09 (2.21, 4.33)	0.90 (0.55, 1.48)
Other	1.93 (0.78, 4.76)	0.86 (0.20, 3.63)
Type 2 diabetes	1.70 (1.23, 2.34)	1.78 (1.17, 2.70)
Cardiovascular disease	1.23 (0.81,1.87)	1.00 (0.55, 1.82)
Cancer history	3.10 (1.52, 6.31)	1.34 (0.46, 3.88)
Hypertension	1.69 (1.20, 2.38)	0.83 (0.53, 1.30)
Kidney disease	2.37 (1.45, 3.89)	1.17 (0.51, 2.66)
Respiratory disease	1.39 (0.89, 2.18)	2.03 (1.26, 3.25)

**TABLE 3:** Stratified analysis exploring risk factors for COVID‐19 death stratified by median age and sex

	Death from COVID‐19 *N* = 5333, *n* deaths = 244			
	Aged ≤43 years *N* = 2681, Deaths = 30	Aged >43 years *N* = 2650, Deaths = 214	Female *N* = 1901, Deaths = 57	Male *N* = 3430, Deaths =187
	Odds ratio (95% CI)	Odds ratio (95% CI)	Odds ratio (95% CI)	Odds ratio (95% CI)
Age (years)	1.11 (1.03, 1.20)	1.04 (1.02, 1.05)	1.05 (1.02, 1.07)	1.05 (1.04, 1.07)
Sex				
Female (ref)	1.00	1.00	–	–
Male	1.27 (0.49, 3.24)	2.15 (1.51, 3.07)	–	–
Ethnicity				
Arab (ref)	1.00	1.00	1.00	1.00
South Asian	3.26 (1.31, 8.12)	2.81 (1.94, 4.07)	3.43 (1.50, 7.84)	3.03 (2.09, 4.40)
Other	6.77 (1.29, 35.62)	1.27 (0.43, 3.71)	3.72 (0.45, 30.63)	1.70 (0.63, 4.56)
Type 2 diabetes	2.70 (0.96, 7.60)	1.59 (1.15, 2.21)	3.57 (1.74, 7.31)	1.40 (0.97, 2.02)
Cardiovascular disease	1.71 (0.24, 12.48)	1.24 (0.81, 1.89)	2.09 (1.00, 4.34)	1.00 (0.60, 1.66)
Cancer history	33.58 (5.85, 192.67)	2.26 (1.06, 4.81)	1.60 (0.36, 7.10)	4.11 (1.76, 9.60)
Hypertension	1.99 (0.68, 5.84)	1.54 (1.10, 2.17)	0.78 (0.38, 1.60)	2.06 (1.41, 3.01)
Kidney disease	18.73 (4.95, 70.84)	1.87 (1.10, 3.18)	2.29 (0.93, 5.64)	2.31 (1.28, 4.17)
Respiratory disease	2.13 (0.57, 7.94)	1.28 (0.79, 2.06)	1.47 (0.74, 3.00)	1.27 (0.69, 2.34)

Estimating PAFs for death, point estimates were 19.6% (95% CI 10.8, 35.6) for type 2 diabetes, 2.7% (95% CI 1.0, 6.8) for cancer, 2.1% (95% CI 0.2, 22.2) for cardiovascular disease, 21.9% (95% CI 11.6, 41.3) for hypertension, 5.0% (95% CI 2.3, 10.8) for kidney disease and 2.4% (95% CI 0.4, 13.5) for respiratory disease.

## 4 DISCUSSION

Although Kuwait has a population with a high prevalence of type 2 diabetes and obesity, death rates from COVID‐19 were comparable to or lower than those reported in other parts of the world.^14^, ^15^ Despite this, type 2 diabetes appears to be an important risk factor for COVID‐19 death. Type 2 diabetes was associated with 70% increased odds of in‐hospital COVID‐19 death, and assuming causality, the PAF for COVID‐19 death caused by type 2 diabetes was 19.6%. Our data suggest that type 2 diabetes is an important, and potentially modifiable, driver of COVID‐19 deaths, and is of particular concern in countries with high prevalence of the condition.

The association between type 2 diabetes and COVID‐19 death observed in the current study (adjusted OR 1.70) is similar to that reported in a national study from England and Wales (adjusted OR 1.80).^16^ This indicates that the condition increases risk by approximately the same proportion in both countries, and therefore, mechanisms linking the condition to increased COVID‐19 severity are probably similar. However, the burden of type 2 diabetes in Kuwait is considerable, and more than 50% of those who died of COVID‐19 in Kuwait had diabetes (in contrast to 31% in the study in England and Wales^16^). This leads to a substantial COVID‐19 PAF associated with diabetes in Kuwait. The association of type 2 diabetes with poor outcomes was explained by poor respiratory function, elevated inflammatory markers, worse renal function and elevated liver enzymes at presentation. This suggests that type 2 diabetes leads to more severe multi‐organ disease at presentation, which then leads to worse outcomes.

Kuwait is in the process of undertaking public health initiatives to lower obesity and diabetes prevalence.^17^ These results support the need for public health campaigns in countries with high type 2 diabetes burden to support weight loss initiatives.^2^, ^18^, ^19^ Whilst we lack trial evidence to prove weight loss lowers risk of severe COVID‐19, weight loss can lead to remission of type 2 diabetes,^20^ and there are many additional health benefits of weight loss.

Limitations of this study include some missingness due to use of real‐world data, although we were able to use multiple imputation. There is also likely to be a burden of undiagnosed type 2 diabetes within this population; our estimates may be underestimates. We acknowledge that admission ‘criteria’ for COVID‐19 are not standardised across times or healthcare practitioners, and therefore, there may be some biases in terms of hospital admission being representative of case severity across time. As these are observational data, caution must be used in making causal inferences.

In summary, these data emphasize the importance of type 2 diabetes as a risk factor for COVID‐19 death in an Arab country, and reiterate the public health importance of lessening diabetes rates in the Middle East. People with diabetes should be considered candidates for vaccine prioritization.

## CONFLICT OF INTEREST

PW has received research grants from Roche Diagnostics, AstraZeneca and Boehringer Ingelheim outside the submitted work, and NS has received grant and personal fees from Boehringer Ingelheim and personal fees from Amgen, AstraZeneca, Eli Lilly, Merck Sharp & Dohme, Novartis, Novo Nordisk, Pfizer and Sanofi outside the submitted work; all authors declare no other relationships or activities that could appear to have influenced the submitted work.

## AUTHOR CONTRIBUTIONS

EA‐O, YH, LA, NV, JAK, DA‐S, AAO, AH and FAM contributed to conception and data acquisition as well as interpretation of the data and critically revised the manuscript for important intellectual content. YH and NV cleaned the clinical data prior to statistical analysis. SVK, SRG, JMRG, CAC‐M and NS contributed to study design, analysis and interpretation of the data and critically revised the manuscript for important intellectual content. RB conducted statistical analyses and with PW contributed to conception and interpretation of the data and wrote the first draft. EA‐O is the guarantor and accepts full responsibility for the work and/or the conduct of the study, had access to the data and controlled the decision to publish.

## ETHICAL APPROVAL

Ethical approval for use of the data was obtained by the standing committee for coordination of health and medical research in the ministry of health in Kuwait (IRB 2020/1400).

## Data Availability

The raw data of the study can be provided upon request with maintenance of confidentiality, privacy and anonymity of the participants and abiding to ethical board regulations.
